# Quantum-Inspired Evolutionary Approach for the Quadratic Assignment Problem

**DOI:** 10.3390/e20100781

**Published:** 2018-10-12

**Authors:** Wojciech Chmiel, Joanna Kwiecień

**Affiliations:** Faculty of Electrical Engineering, Automatics, Computer Science and Biomedical Engineering, AGH University of Science and Technology, al. Mickiewicza 30, 30-059 Kraków, Poland

**Keywords:** quadratic assignment problem, quantum-genetic algorithm, quantum computing

## Abstract

The paper focuses on the opportunity of the application of the quantum-inspired evolutionary algorithm for determining minimal costs of the assignment in the quadratic assignment problem. The idea behind the paper is to present how the algorithm has to be adapted to this problem, including crossover and mutation operators and introducing quantum principles in particular procedures. The results have shown that the performance of our approach in terms of converging to the best solutions is satisfactory. Moreover, we have presented the results of the selected parameters of the approach on the quality of the obtained solutions.

## 1. Introduction

The quadratic assignment problem (QAP) is one of the most interesting and difficult combinatorial optimization problem. Due to its popularity, many publications have focused on the QAP problem to search for methods that are sufficient for practical applications. Some studies have focused on the applicability of the QAP to the solution of many various problems. There exist several problems which are specializations of this problem, like: graph partitioning and maximum clique problem, travelling salesman problem, graph isomorphism and graph packing problem [1]. The QAP problem has been shown to be NP-*hard* [2], hence several approaches have been used to solve this problem. Intensive studies on quadratic assignment problems produced many algorithms over the last few decades. For a survey on these methods, see [3,4]. It should be mentioned that the performance of the methods for solving the quadratic assignment problems depends on the complexity of the problems. Due to the computational complexity of the QAPs, exact methods can solve relatively small-sized instances from the QAP benchmark library (QAPLIB) with up to 30 locations. Therefore, to obtain near-optimal solutions, various heuristic and metaheuristic approaches have been developed, such as tabu search [5,6,7], simulated annealing [8,9], scatter search or swarm algorithms including ant colony optimization [10], particle swarm optimization [11,12] and bees algorithm [13,14]. One of the initiatives followed by many researchers is using evolutionary algorithms for solving quadratic assignment problems [3,15,16,17,18]. Although these algorithms do not ensure obtaining optimum solutions, they produce good results in a reasonable computation time.

In this paper we focus on the quantum-inspired evolutionary algorithm (QIEA) that draws inspiration from evolutionary computing and quantum computing. It is worth mentioning that the harnessing of quantum computing to the study of various problems can take two forms. One may choose to adapt some principles of quantum computing in the classical existing approaches. Alternatively, a quantum mechanical hardware may be sought via the studies.

In recent years quantum-inspired algorithms have received growing attention. Many researchers have presented various quantum-inspired evolutionary algorithms to solve many optimization problems with success, including image processing [19], network design problems [20,21], scheduling problems [22,23,24,25], real and reactive power dispatch [26], parameter estimation of chaotic systems [27], parameter selection for support vector machines [28], community detection on CUDA-enabled GPUs [29] etc.

Below we provide a brief summary of different methods for solving QAP. In many cases, evolutionary algorithms and their hybridizations solved different combinatorial optimization problems quite successfully. In [30,31] a hybrid genetic algorithm and its variants for solving the quadratic assignment problem (QAP) are studied. Benlic et al. [32] obtained very promising results using a new variant of the memetic algorithm for QAP, where a solution created by the crossover operator is improved using the local optimisation procedure BLS (breakout local search) or by the adaptive mutation procedure. Lalla-Ruiz et al. [33] proposed the hybrid biased random key genetic algorithm for the QAP problem, where the chromosomes are random key vectors. In turn, Luong et al. [34] proposed the multi-objective gene-pool optimal mixing evolutionary algorithm (MO-GOMEA) with the automatic selection of the algorithm parameters. The use of Sule’s Method and genetic algorithms in a real industry application formulated as the QAP was proposed in [35]. It should be mentioned that extensive research was carried out on developing various specific modifications of particular components of evolutionary algorithms to increase the EA efficiency, including crossover schemes [16,36,37] or replacement strategies of the population [25].

There are many algorithms, in which the nature inspired approach is combined with the methods from different domains. Metlicka et al. [38] proposed the chaos driven discrete artificial bee colony (CDABC) algorithm with the pseudo-random number generator based on the chaos pseudo-number generators using the chaos maps. The distributed multi-agent optimization model for QAP (MAOM-QAP) was designed by Sghir et al. [39], where the cooperating agents, such as decision-maker, local search, crossover and perturbation agents, were included in the intensification or diversification of the search process.

In turn, Duman et al. [40] proposed the algorithm based on the phenomenon of the migrating birds. The algorithm explores the proportionally smaller number of neighbourhoods for solution (birds) at the back of the V bird formation. Olivera et al. [41] proposed the population-based ant colony optimization algorithm (P-ACO) with the original pheromone update algorithm. Hafiz et al. [42] proposed the PSO algorithm for the QAP, which introduces the probabilistic learning process based on the identifying beneficial assignment of the facility to a particular location. Dokeroglu et al. [43] proposed a hyper-meta-heuristics for QAP, where well-known heuristics such as the simulated annealing (SA), the robust tabu search (RTS), the ant colony optimization (ACO) and the breakout local search (BLS) cooperate in the parallel.

Moreover, other approaches were tested. For example, Tasgetiren et al. [44] proposed for QAP the variable block insertion heuristic in the single-solution version (VBIH) and in the populated version (PVBIH). Yuan et al. in [45] studied the evolutionary multitasking of the permutation-based combinatorial optimization problems (PCOPs) and proposed a new unified representation and the new survivor selection procedure. The BLS-OpenMP algorithm was proposed by Aksan et al. [46]. In this algorithm local search heuristic uses the Levenshtein distance metric for checking similarity of the new starting points to the previously explored solution of QAP. Acan et al. [47] proposed the heuristic, where two populations of solutions act as a short-term and a long-term memory and cooperate within the great deluge algorithm (GDA) which is similar to the simulated annealing (SA) algorithm where the level-based acceptance is dynamically adjusted.

Theoretical developments of quantum-inspired evolutionary algorithms and applications of their different types are presented in [48].

Although there are studies on the individual topics of the quadratic assignment problem and quantum-inspired evolutionary algorithms, we have found none that covers both of these two topics. The purpose of this paper is to demonstrate that QIEA for solving QAP is possible through the correct design of particular procedures. Therefore, we assumed that QIEA can be applied after its modification, concerning a representation of solutions to the proper choice of crossover operators and quantum gates. We incorporate a rich set of examples to illustrate the application of the different operators in our approach and to show that appropriate modifications are needed to ensure the admissibility of solutions and efficiency of our approach.

Moreover, to introduce the basics of the quantum computing and the quadratic assignment problem, we have endeavoured to provide some in-depth quantum-inspired evolutionary algorithm for solving QAPs. The organization of this paper is as follows: Section 2 provides an overview introducing QAP, with some examples of its applications. Due to considerable importance of the quadratic assignment problem, this section briefly describes the Koopmans–Beckmann model. Section 3 gives more insight into the quantum-inspired evolutionary algorithms. In order to cope with the application of this algorithm to solve the quadratic assignment problem, we present some adaptations of the algorithm such as an appropriate representation of a solution, the crossover operators, the involvement of a quantum gate, the mutation procedure and the local search (2-opt). In Section 4, we provide the results of conducted experiments with respect to our approach performance on selected instances. Much work focuses on the impact of various parameter settings. We tested the impact of the 2-opt probability and the gate’s occurrence on the quality of the obtained solutions. The final section regards a discussion of the results and summarizes the conclusions.

## 2. Quadratic Assignment Problem

The QAP problem was introduced by Koopmans and Beckmann in 1957 as a mathematical model for the assignment of a set of economic activities to a set of locations, with taking into account the distance and flow between the facilities and the costs of placing a facility in a specific location.

Let us now define the three non-negative matrices $D={\left[d_{ij}\right]}_{n\times n}$, $F={\left[f_{ij}\right]}_{n\times n}$, $B={\left[b_{ij}\right]}_{n\times n}$ for the given set N=1,…,n and permutation π as the solution to the QAP problem. Thus, π(i)∈N(i=1,…,n) corresponds to the index of the facility and the set *N* is the set of the location indexes to which the facilities are assigned.

Formally, the QAP problem can be formulated as follows: given distances between locations (matrix D), the flow (weight, number of connections) between pairs of facilities (matrix F), and the assignment cost of the facility *m* to the position *n* (matrix B), which in most cases is omitted. The solution of QAP (also denoted as QAP(F,D,B)) can be shown with the permutation form π=(π(1),…,π(n)) of the set of *n* elements (facilities). The aim of solving the Koopmans–Beckmann model is to find the permutation $\pi^{*}$ in the set of permutations Π, so that:

(1) $$f\left(\pi^{*}\right)=\min_{\pi \in \Pi }f\left(\pi \right)$$

where

(2) $$f\left(\pi \right)=\sum_{i=1}^{n}\sum_{j=1}^{n}f_{ij}d_{\pi (i)\pi (j)}.$$

The aim is to minimize the objective function f(π) which describes a global cost assignment of *n* facilities to *n* locations. Π is the set of permutations on the set *N*. In most cases the matrix D is symmetric (distance $d_{ij}$ between two locations *i* and *j* is the same as between *j* and *i*). Matrix *F* is symmetric if $f_{ij}$ is regarded as connections.

As mentioned in the previous section, Sahni and Gonzales [2] proved that QAP is strongly NP-*hard* by showing that the existence of a polynomial time algorithm for solving QAPs with the entries of the coefficient matrices belonging to {0,1,2} implies the existence of a polynomial time algorithm for an NP-*hard* decision problem (the Hamiltonian cycle problem).

Many researchers discussed and examined the quadratic assignment problem in respect of its practical use. They proposed its application for solving various real problems e.g., hospital lay-out [49], campus planning model [50], backboard wiring problem [51], and so on. Reviews on some practical applications of QAPs can be found in [4].

## 3. Quantum-Inspired Evolutionary Algorithms

By combining quantum computing with an evolutionary algorithm, Han and Kim [52] proposed the first quantum-inspired evolutionary algorithm (QIEA) with quantum coding of chromosomes and a quantum rotation gate as a variation operator to increase obtaining better solutions. Apart from rotation gates, more various quantum gates such as the NOT, AND, OR, NAND, Hadamard, can be applied to modify the state of a qubit [53]. We do not discuss all the gates for changing the probabilistic distribution of each individual. The interested reader is referred to the original literature [53]. Moreover, a comprehensive survey of studies over quantum-inspired evolutionary algorithms is provided in [48]. In this section, firstly we briefly describe the principles of quantum computing, the difference between the coding used for the standard and quantum algorithms, and the main components of our approach.

### 3.1. Principles of Quantum Computing: Quantum Bit and Quantum Gate

The quantum-inspired evolutionary algorithms use quantum bits (qubits, Q-bits) to represent individuals, quantum gates employed to operate on the Q-bits to create the next generation (offspring) by employing an observation process to connect the Q-bit representation with the optimization variables. The Q-bit individual can describe a linear superposition of the basis states in a search space probabilistically and its representation maintains the population diversity. It is represented by a vector in the Hilbert space with the basis states |0〉 and |1〉.

Therefore, the superposition |Ψ〉 of the qubit is represented as follows:

(3) |Ψ〉=α|0〉+β|1〉,

where α and β are numbers that specify the probability amplitudes of the corresponding states and satisfy the normalization condition ${\left|\alpha \right|}^{2}+{\left|\beta \right|}^{2}=1$.

Hence, the values ${\left|\alpha \right|}^{2}$ and ${\left|\beta \right|}^{2}$ give the probabilities that the Q-bit will render the ‘0’ or ‘1’ states, respectively. Generally speaking, a qubit is the smallest unit of information and is represented by a pair of numbers ${\left[\alpha \beta \right]}^{T}$. If the qubit individual *q* is defined as a string of the *n* qubits, a system has *n* Q-bits and expresses the $2^{n}$ states as follows:

(4) $$q=\left[\begin{array}{ccccc} \alpha_{1} & \alpha_{2} & \alpha_{3} & \cdots & \alpha_{n}\\ \beta_{1} & \beta_{2} & \beta_{3} & \cdots & \beta_{n} \end{array}\right].$$

**Figure 1:**
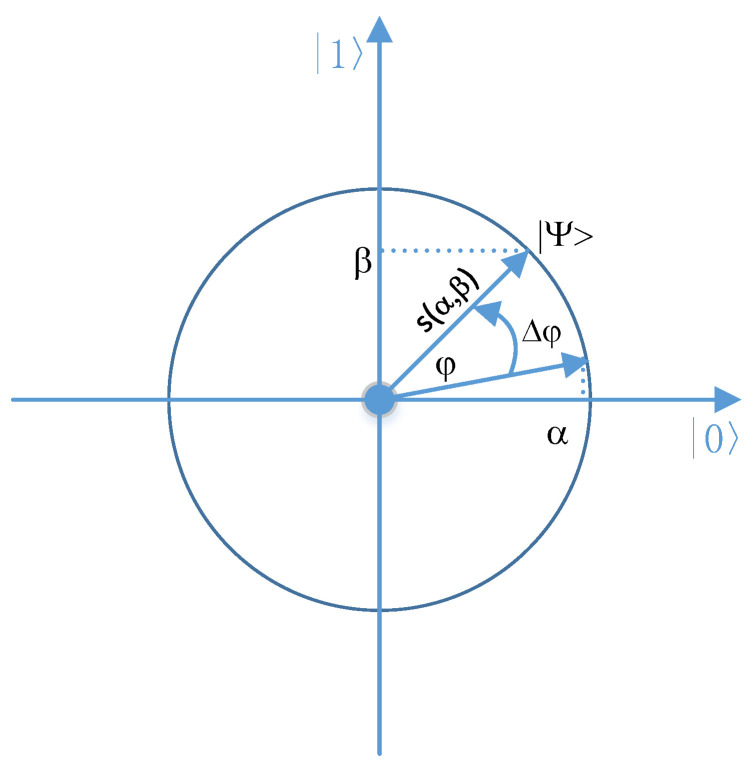
Rotation Q-gate geometric interpretation.

The $i^{th}$ Q-bit is updated by applying the following quantum rotation gate (Q-gate) [53]:

(5) $$G\left(\varphi \right)=\left[\begin{array}{cc} cos\varphi & -sin\varphi \\ sin\varphi & cos\varphi \end{array}\right],$$

where φ is the Q-gate rotation angle defined as:

(6) φ=s(α,β)Δφ

and s(α,β) and Δφ are the direction of quantum gates rotation (the sign of φ) and the magnitude of rotation angle of φ, respectively. The rotation Q-gate quantum operator is presented in Figure 1.

The direction and the rotation angle are given in the look-up table (Table 1) according to [54], where f(x) and f(b) represent the fitness of the current chromosome and the fitness of the best individual, respectively.

However, it should be remembered that the rotation Q-gate quantum operator has a disadvantage. The values in the look-up table can affect the algorithm performance. Note that the rotation angle has an effect on the convergence speed. One way to avoid this problem is to use an adaptive strategy as was shown in [55].

### 3.2. Quantum Evolution for the Quadratic Assignment Problem Algorithm

The proposed algorithm was built on the principles of the genetic algorithm and quantum mechanics. As we know, the efficient mechanisms of genetic algorithms make them useful for different combinatorial problems. The algorithm goes up to the pseudo-optimal solution by the population update based on the selection, crossover and mutation operators. By combining these genetic operators, we can implement various genetic algorithms. For surveys of the crossover operators and their investigation within genetic algorithms for solving QAPs, see [56].

The proposed Quantum Evolutionary Algorithm for QAP problem ($Q^{2}\mathrm{APA}$) algorithm uses the solution representation based on the qubits. It employs several types of the pseudo-genetic operators which operate on the permutation form of the solution. Before using this operator, the qubit form of the solution is decoded to the form of the permutation. The algorithm uses the following crossover operators designed for the permutation solution representation: PMX (partially matched crossover), OX (order crossover) and CX (cycle crossover).

**Figure 2:**
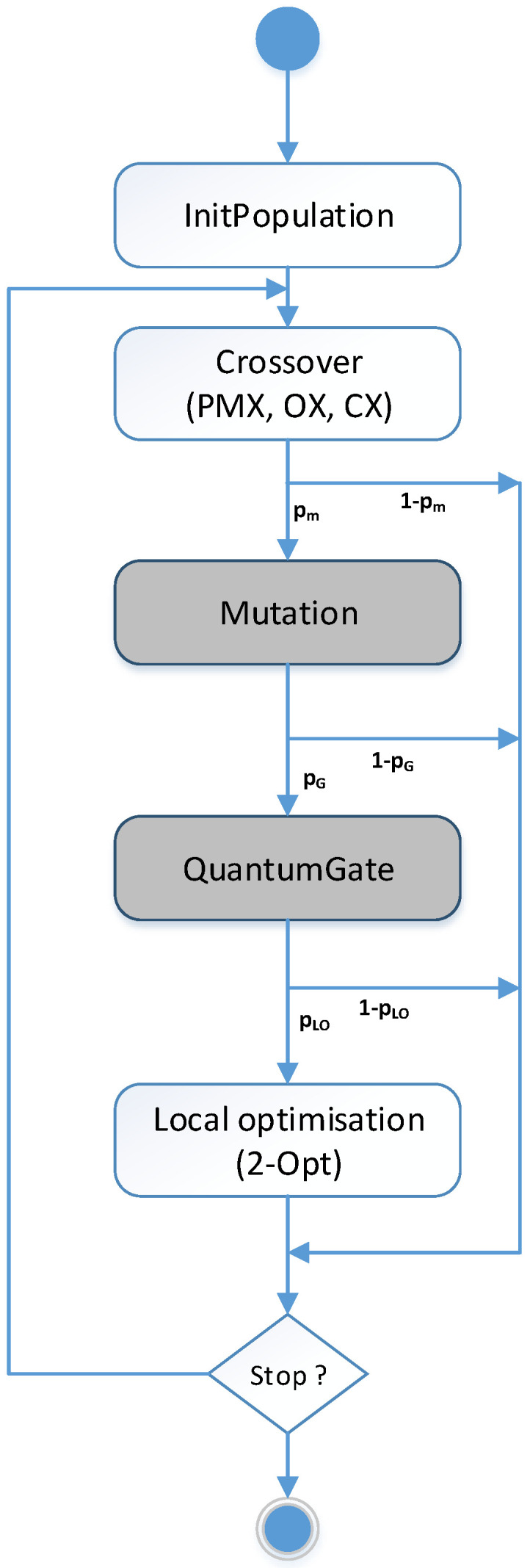
$Q^{2}\mathrm{APA}$ algorithm flow.

The general structure of the $Q^{2}\mathrm{APA}$ approach is illustrated in Figure 2. The implemented procedures which use the qubit representation of the permutation are marked with the gray colour. The main components of our approach are presented in detail in the following sections.

#### 3.2.1. Algorithm Initialization and Selection

The *InitPopulation* procedure creates λ solutions in the form (4), where $\alpha_{k},\beta_{k}$ are real values generated randomly with the uniform distribution and $k=1,2,\ldots ,(\log_{2}n+1)n$, where *n* denotes the problem size. In the algorithm, the selection procedure can take two forms. One may choose to adopt the roulette wheel method. Therefore, there will be more chromosomes that have the lower objective function value in the new generated population. For each chromosome in the population the fitness value $f(\pi_{i})$ of permutation $\pi_{i}$ is given as the difference between the worst solution obtained in the population and the value of the objective function. Alternatively, the ranking method may be sought. The solutions in the current generation are ranked in ascending order according to the value of the objective function. Then, based on the ranking, a function is built, the value of which determines the probability of choosing a given solution during the selection. There are two basic variants of these functions—the linear version and the non-linear one. In the paper, we assume that the probability of choosing a given solution $p(\pi_{i})$ to be a parent is based on the linear version of the ranking:

(7) $$\forall i\in \left\{1,\cdots ,\lambda \right\}:rank\left(\pi_{i}\right)=i\Leftrightarrow \forall j\in \left\{1,\cdots ,\lambda \right\}f\left(\pi_{i}\right)<f\left(\pi_{j}\right)$$

(8) $$p\left(\pi_{i}\right)=\frac{1}{\lambda }\left(\eta_{max}-\left(\eta_{max}-\eta_{min}\right)\frac{i-1}{\lambda -1}\right)$$

where $\eta_{max}$ defines decrease in probability of the selection as the parent if the ranking of the solutions decreases and $\eta_{min}=2-\eta_{max},1\leq \eta_{max}\leq 2$.

#### 3.2.2. Crossover Process

All solutions in the population are processed by the crossover operator. The set of the crossover operators contains a special type of the operators designed to the permutation crossover, such as: PMX, OX, CX. On the basis of the crossover of the two permutations (parents) the two valid permutations (siblings) can be obtained [57]. The first well-known crossover that has been applied in our approach is the PMX operator. It starts with a random choice of two crossover points in the parent permutations (the same in both parents). Genes located in such a part of the permutations are swapped (by mapping) between the parents. Other positions are rewritten if they are not present in the offspring permutations. If the conflict occurs, genes are replaced with the use of a mapping relationship.

While creating new solutions, the order crossover (OX) assumes a randomly selection of two cut points in two parent permutations and preserves the order of genes. The selected part of one parent chromosome between these points is copied to the offspring. The unassigned positions are sequentially supplemented and taken from the other parent in order, starting from the first gene after the second cut point. After reaching the end of the parent permutation, one performs additional assignments from the beginning of this parent until all genes have been considered.

During the CX operator all offspring genes are taken either from the first or second parent. All genes found in the same positions in both parents are assigned to the child’s corresponding locations. Starting from the first or the randomly selected location that has not be included in the offspring yet, an element is randomly selected from both parents. Then, additional assignments are made to avoid random assignments. The next unassigned location is processed in the same way until all locations are included.

#### 3.2.3. Mutation Procedure

Each solution in the population is represented by the tuple $S_{ij}=\left\{P_{ij},Q_{ij}\right\}$, where $Q_{ij}$ is the qubit representation of the solution while $P_{ij}$ is the observable state of the qubit, a permutation. The indexes *i* and *j* define the index of the population (iteration) and the index of solution in the population, respectively. Before application of the mutation operator, the solution has to be moved from the superposition state ($Q_{ij}$) to the observable state ($P_{ij}$) using the *ObservableState* procedure (see Algorithm 1).

**Algorithm 1** ObservableState.**Require:**$Q_{ij}$, *n*. **Step 1. For each**$k\in [1,\ldots ,\left(\log_{2}n+1\right)n]$ generate uniformly value ρ∈[0,1]
**If**${\rho <|\alpha |}^{2}$**then**$q_{k}=1$**else**$q_{k}=0$ and obtain the binary string: $\left[q_{1}q_{2},\cdots q_{(\log_{2}n+1)n}\right]$.**For each**i∈[1,⋯n] translate the binary substring $\left[q_{i+1}q_{i+2}\cdots q_{i+(\log_{2}n+1)n}\right]$ to the decimal value and obtain the string of the decimal number $S_{d}=\left[d_{1}d_{2}\cdots d_{n}\right]$.**Step 2.** Replace each number $d_{i}$ in this way that the $S_{d}$ takes a permutation form. **For**
i=1
**to**
*n*
**do**:Find the set of smallest, not marked, elements in the string $S_{d}=\left[d_{1}d_{2}\ldots d_{n}\right]$.Replace the smallest element from the left side of the string - $d_{k}$, by the value *i* and set the value *i* at the position *k* as marked.**Return** string $S_{d}$ which has a permutation form.

The mutation operator which generates a random permutation in the Hamming distance equals 2 by swapping two randomly selected elements in the permutation. It allows preventing the $Q^{2}\mathrm{APA}$ algorithm from being trapped into the local optimum. To control exploration properties of our approach, the Q-gate operator using the quantum solution representation is used.

All the solutions generated using the Q-gate operator are improved using the *2-opt* local optimization procedure which effectively examines $\frac{n(n-1)}{2}+1$ neighbourhood solutions. The mutation operator changes randomly a binary qubit value. Afterwards, this mutated solution in the qubit form is processed by the Rotation Q-Gate operator. In this paper we propose to control the size of the rotation angle of Q-gate. We assume that the angle φ is defined as a variable related to the generation number *i* (Figure 2). For example, the geometric reduction of the Q-gate angle expressed as $\varphi \cdot \delta^{i}$ can be used, where δ<1. In the experiment, depending on the algorithm settings, the modified or original values from the look-up table are used (Table 1). Below, the code fragments in C# responsible for the quantum mutation and the standard rotation gate (according to Table 1) are presented.



 public class MutationOperator : IMutationOperator

 {

   private static MersenneTwister rand = new MersenneTwister();

   public double MutationProbability { get; set; }

   public MutationOperator(double probability = 0.0) {this.MutationProbability = probability;}

   public ISolution Execute(IPopulation population)

   {

      double ifMutate;

      Solution solToReturn = null;

      foreach(Solution sol in population){

         ifMutate = rand.NextDouble();

        if(ifMutate <= this.MutationProbability){

         int selectedChromosome = rand.Next(0, sol.Size - 1);

            int bitsInChromosome = (int)(Math.Log(sol.Size, 2.0) + 1);

            int selectedQbit = rand.Next(0, bitsInChromosome - 1);

            sol[selectedChromosome][selectedQbit].ExecuteNotGate();

            sol.toPermutation();

            solToReturn = new Solution(sol);

            break;

         }

       }

       return solToReturn;

    }

}

....

 

public class RotationGateOperator : IEvolutionaryOperator

{

...
   public void ExecuteOriginal(IPopulation population, Solution best, double alpha)   {      double theta;      double alphaTimesBeta;      double angle = 0.0;      double sign = 0.0;      this.solSize = best.Size;      this.bitsInSol = (int)(Math.Log(this.solSize, 2.0) + 1);      Solution prevSolution = null;
 
      foreach (Solution sol in population){       if (sol.Goal >best.Goal){         prevSolution  = new Solution(sol);         for (int i = 0; i < this.solSize; i++){            for (int j = 0; j < this.bitsInSol; j++){               theta = 0.0;               alphaTimesBeta = sol[i][j].Alpha * sol[i][j].Beta;               angle = 0.0;               sign = 0.0;               if (sol[i][j].ObservedState == 1 &&  best[i][j].ObservedState == 0){                  if (alphaTimesBeta > 0.0) sign = -1.0;                  else if (alphaTimesBeta < 0.0) sign = 1.0;                  else if (sol[i][j].Alpha == 0.0){                     double d = rand.NextDouble();                     if (d > 0.5) sign = 1;                     else sign = -1.0;                  }                  angle = 0.5 * Math.PI;               }               else if (sol[i][j].ObservedState == 1 && best[i][j].ObservedState == 1){                  if (alphaTimesBeta > 0.0) sign = 1.0;                  else if (alphaTimesBeta < 0.0) sign = -1.0;                  else if (sol[i][j].Beta == 0.0){                     double d = rand.NextDouble();                     if (d > 0.5) sign = 1.0;                     else sign = -1.0;                  }                  angle = 0.2 * Math.PI;               }               theta = angle * sign;               if (theta != 0.0) sol[i][j].ExecuteRotationGate(theta*alpha);            }         }         sol.toPermutation();         if (sol.Goal > prevSolution.Goal) sol.BestSolution = prevSolution;      }   } }
}



#### 3.2.4. Detailed $Q^{2}\mathrm{APA}$ Algorithm Flow

Once an initial population of quantum chromosomes is created, these are used to create a population of permutations. It should be mentioned that each solution is evaluated to give a level of its fitness. Upon their selection, the offspring solutions are formed by the multiple operators: crossover, mutation, Q-gate and 2-opt. The qubit state update is performed if the solution has been subject to changes resulting from the operation of the quantum gate operator or for this solution the occurrence of the conditions for the mutation has been met. However, after performing a crossover operation, the state of the qubits of the child solutions is not evaluated. Note that such an assessment (mostly in the early iterations of the algorithm) would cause that the solutions obtained through the crossover could lose the information obtained from the parents’ solutions. Q-bit individuals are modified by applying the rotation Q-gate with probability $p_{m}$. Then, the state of each qubit is checked in the best solution and compared to the state of the corresponding qubit in the solution obtained by the quantum gate. In the next step, the quantum chromosomes (the set $Q^{\prime }$) are converted to permutations (the set $\Pi^{\prime }$) by using Algorithm 1 and they are improved using the *2-opt* procedure with the probability $p_{LO}$ (see Figure 2). Without the use of a quantum gate, the quantum idea of the algorithm is then manifested only during the creation of the initial population and the mutation.

**Figure 3:**
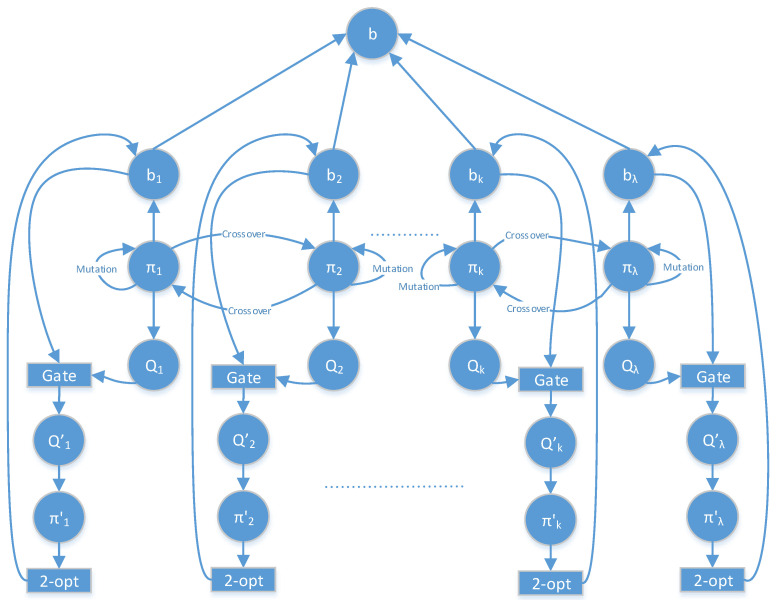
Detailed $Q^{2}\mathrm{APA}$ algorithm flow.

Taking into account the described procedures, our algorithm’s flow is shown in Figure 3, where *b* denotes the best solution found by the $Q^{2}\mathrm{APA}$ algorithm during the evaluation process of the population, and $b_{1},\ldots .,b_{\lambda }$ represent solutions for their two forms (the permutation and quantum), so $b_{i}=\left\{\pi_{i},Q_{i}\right\}$. It is important to note that, at the beginning, the permutation forms of the solutions are evaluated using the mutation and the crossover operators. These operators do not affect the quantum state of the parents. On the basis of the offspring’s permutation, the quantum representation of the solutions is created. When analyzing Figure 3, one can conclude that the solutions in the population are randomly changed (with the predefined probability) using the quantum rotation gate or/and the *2-opt* procedure.

## 4. Experiments and Results

The aim of the proposed experiments is to test the possibility of using the quantum representation to improve the results obtained by the evolutionary algorithm for the QAP problem. We evaluate the performance of the $Q^{2}\mathrm{APA}$ algorithm by testing it on the well-established benchmark instances from the QAPLIB, whose size is indicated in the instance name. Therefore, we tested it through a number of experiments on the QAP instances with the known reference solutions. As we know, the QAPLIB contains various instances of the QAPs, which stem from real-life problems (architecture, computer science, etc.) and the instances generated for testing problems with the special properties. The solution quality was taken into account to assess the performance of the algorithm. Therefore, we conducted many runs of the $Q^{2}\mathrm{APA}$ on the 37 instances of varying complexity. For each test instance, we assume the same setting of parameters through 10 independent runs of the algorithm. For this purpose, we obtained the relative deviation (Dev) of the best found objective value ($f_{best}$, the best value of ten independent runs) by our approach from the best known value ($f_{ref}$) reported in the QAPLIB as follows:

(9) $$Dev=\frac{f_{best}-f_{ref}}{f_{ref}}\times 100\%$$

Various crossover operators, gates and 2-opt probabilities will be discussed with a view to characterizing the results obtained from their application. As we know by choosing different values of parameters, there are different results one can obtain. Therefore, a question is if there is any particular value better than the others. In what follows, we will restrict attention to characterizing the best values of the $Q^{2}\mathrm{APA}$ parameters resulting from their application.

The algorithm was implemented in C# programming language using Windows 10 operating system. The computer parameters used for calculations are presented in Table 2.

### 4.1. Impact of Different Crossover Operators

The first series of the experiments aimed at determining the best operator in the crossover process among the three operators: PMX, CX, OX (see Section 3.2.2). These crossover operators for creating offspring were tested on two groups of the instances stored in the QAPLIB. It should be noted that these experiments were conducted using other fixed parameters during all the iterations. In the experiments, the stopping criterion was the maximum number of the iterations which equals 1000. We used the mutation rate equalling 0.01, the probability crossover equalling 0.7, the population size equalling 350 individuals, the probability of 2-opt equalling 0.2. We applied the roulette and the ranking method for the population selection. We have found that the $Q^{2}\mathrm{APA}$ with the PMX operator and the ranking method obtains the best results. Therefore, we recommend the choice of the PMX operator in other instances and the next experiments.

### 4.2. Impact of 2-opt Probability

Furthermore, we investigated what value of the 2-opt’ probability was correct. In the tests, we assumed the following fixed parameters: the maximum number of iterations = 10,000, the mutation rate equalling 0.01, the probability crossover (PMX) equalling 0.7, the gate’s probability equalling 0.7, the population size equalling 350 individuals. To determine the best value of the probability of the *2-opt* procedure we conducted many experiments to assess the performance of the $Q^{2}\mathrm{APA}$ algorithm. We used the BUR26 problems taken from the QAPLIB library to verify the impact of this parameter on the results of the $Q^{2}\mathrm{APA}$ algorithm. The relative deviations (Dev) of the found solutions from the reference solutions for the different values of the *2-opt* probability ($p_{LO}$) are summarized in Table 3. On the basis of these experiments, it should be noted that for instances BUR26, the best probability value is 0.4.

### 4.3. Impact of Gate’s Probability

**Figure 4:**
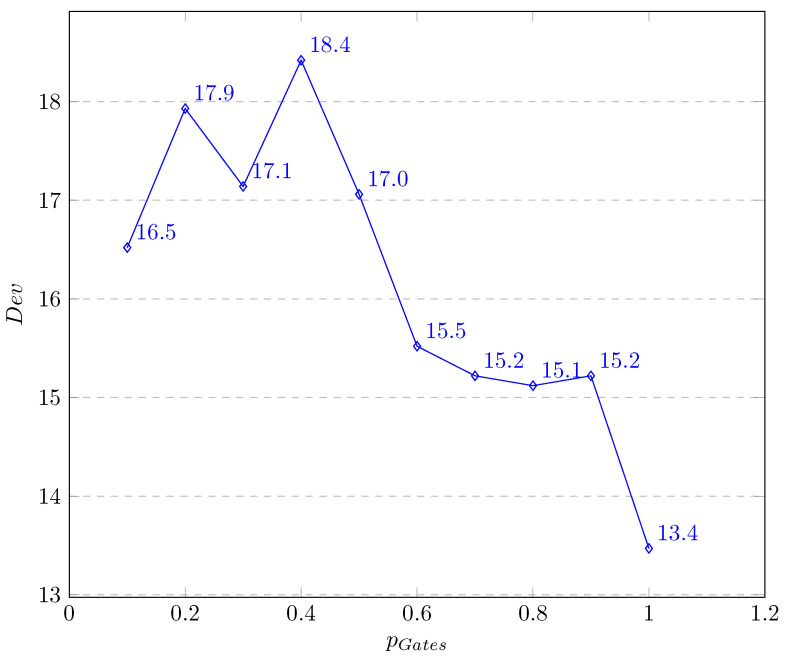
Influence of gate’s probability.

During experiments we were interested in testing several values of the gate’s probability and finding the best one. Therefore, only this parameter varies while the others are fixed. In the experiments, each run of the $Q^{2}\mathrm{APA}$ was terminated after 10,000 iterations. We used the mutation rate equalling 0.01, the probability crossover equalling 0.7, the population size equalling 350 individuals, the probability of the 2-opt equalling 0.4, and the ranking method for the population selection. The results of the $Q^{2}\mathrm{APA}$ for all the considered instances (over the 10 consecutive runs for each instance), relating to the different values of the gate’s probability are presented in Figure 4. One can see that increasing probability of the gate’s occurrence (the value of the horizontal axis) can reduce Dev results.

Table 4 shows the selected results for one value of the gate’s probability (0.8). The table is organized as follows: the first column contains the instance’s name, the second column contains reference solutions, the third column presents the best initial solution. Two next columns $f_{best}$ and $f_{best_{avr}}$ display the best solution and the average solution of the 10 independent runs found by the $Q^{2}\mathrm{APA}$. The iterations with the best solution and the average number of the iterations are given in the sixth and seventh columns. $T_{avr}$ defines the average execution time in milliseconds of the 10 runs of the algorithm. The last column contains the information on the relative deviation.

On the basis of the relative deviations of the solutions obtained (Table 4), it should be noted that for the four test instances (from the ESC* group) the $Q^{2}\mathrm{APA}$ finds the reference solutions. In three cases (ESC32E, ESC32F, ESC32G), the reference solution was found through all the runs of the algorithm in the small numbers of the iterations. Note for the ESC32E and the EC32F that the optimal solutions were found in the first iteration. In the case of instances from the BUR* and LIPA*A groups the solutions are close to the best known solutions. For the analysed instances, the mean value of Dev ($Dev_{av}$) equals 15.12%. It leads to the conclusion that the results obtained with these fixed settings of the parameters for all instances are not good enough.

### 4.4. Best Results

In this section, the best results obtained to gain the overview on differences in the performance between the different parameter settings in the $Q^{2}\mathrm{APA}$ algorithm were presented. These values can be vital to affect the convergence. For example, the experiments in the previous section indicated the influence of the probability of the gate’s occurrence on the obtained solutions. Therefore, the different values of this parameter and the crossover probabilities through the experiments were used. All the experiments conducted in the context of the effect of the various crossover probabilities and the probability of the gate’s occurrence are summarized in Table 5.

Implementation of the variable parameter settings in the $Q^{2}\mathrm{APA}$ algorithm improves its efficiency, but does not guarantee that the well-known reference solutions will be reached. It should be noted that for the 13 instances we obtained better results (cf. Table 4). For all the analysed instances, the mean value of Dev does not exceed 6.45%.

**Figure 5:**
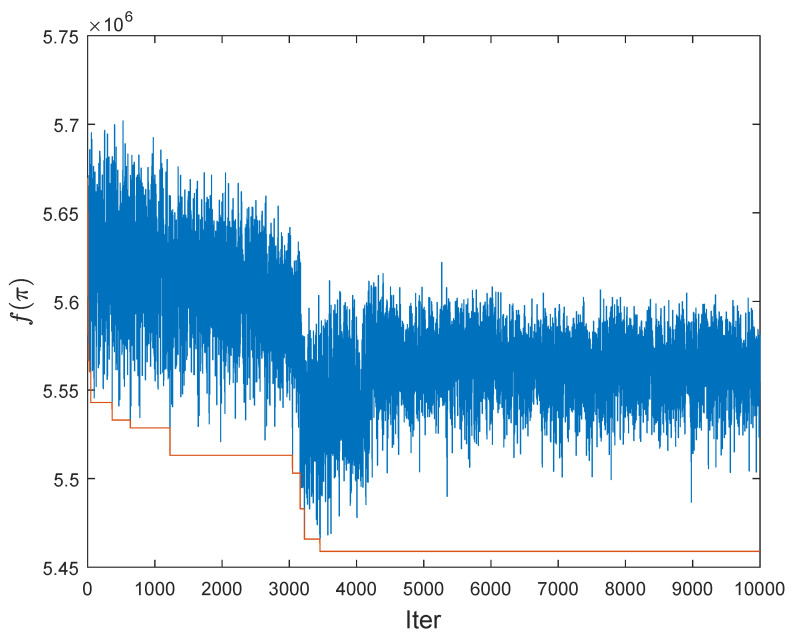
Best run of $Q^{2}\mathrm{APA}$ for BUR26A (gate’s probability equals 0.7).

The selected course of the optimization process for the $Q^{2}\mathrm{APA}$ algorithm for the BUR26A instance is illustrated in Figure 5. It shows the dependency between the objective function value for the best solution and the iteration number. The algorithm during optimization process improves the objective function value, no doubt.

**Figure 6:**
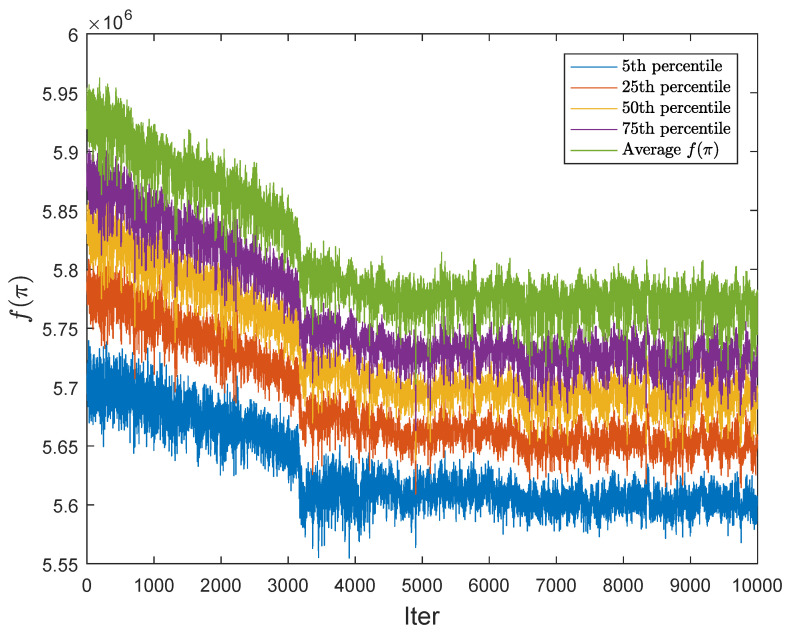
Selected percentiles for BUR26A (gate’s probability equals 0.7).

Figure 6 shows the values of the median and the 5th, 25th, and 75th percentiles for the iterations. One can see a clear drop in all the values in the subsequent iterations. It should be noted that the variability of the results obtained is inevitable, because $Q^{2}\mathrm{APA}$ is a stochastic algorithm.

**Figure 7:**
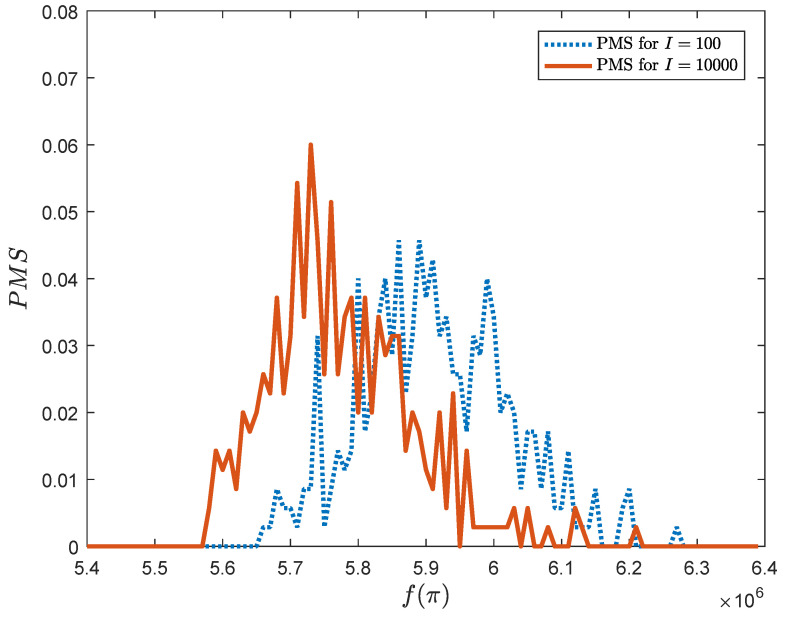
Probability mass function for BUR26A, for iteration I = 100 and I = 10,000 (gate’s probability equals 0.7).

Figure 7 shows how the probability distribution (the probability mass function PMF) changed during the optimization process. In both cases (I=100,I=10,000) the optimization process starts with the normal distribution with the mean value about $5.9\times 10^{6}$. In the subsequent iterations the maximum of the PMF grows and moves towards the smaller values of the objective function, keeping the population relatively diverse.

The results obtained by the $Q^{2}\mathrm{APA}$ algorithm are compared with the results obtained by the two other algorithms: the standard genetic algorithm (GA) and the genetic algorithm which cooperates with the simulation annealing algorithm (GASA) [58] for the same set of the QAP problem instances (see Table 5). The results of the $Q^{2}\mathrm{APA}$ are much worse because the number of iterations executed by the GA and GASA is higher (from two to six times) than the number of iterations executed by the $Q^{2}\mathrm{APA}$ in our experiments. As we mentioned, we tested the possibility of using the quantum representation in the construction of the approximation algorithms. It seems that the quantum representation can be successfully used in the scenarios where the effective exploration of the search space is required.

## 5. Conclusions

This paper proposed the $Q^{2}\mathrm{APA}$, the purpose of which was to solve the quadratic assignment problem using the quantum paradigm. We merely present a study about the quality of the obtained solutions with our adaptations. It should be mentioned that many valuable results were obtained with experiments involving various settings of the algorithm control parameters. Beside these, we involved the 2-opt for modification of solutions. It should be mentioned that the experiments were conducted to determine how the particular control parameter values influenced the performance of the implemented algorithm. As we know, a big value of the rotation angle can lead to premature convergence. In contrast, a small value can increase the chance of finding a better solution, but the convergence time increases.

Our experiments show that selecting the parameters’ values can influence the results. The results might be better in the case of using their optimal settings, so we plan to take into account methods for parameter control and tunning. The implemented method does not include entropy that provides information regarding the spread of the solutions’ values of objective function. Therefore, entropy adapted for use in combinatorial optimization problems including the QAP could improve diversity population, especially at later stage of the algorithm. The future research will be devoted to combining various local search procedures with the described framework. Moreover, one possibility for future work is the performance of GPU implementation for the described algorithm and using a larger number of the QAP instances with bigger size in tests.

## Figures and Tables

**Table 1:** Parameters in look-up table.

*x*	*b*	f(x)<f(b)	Δφ	s(α,β)			
				α·β>0	α·β<0	α=0	β=0
0	0	False	0.2π	0	0	0	0
0	0	True	0	0	0	0	0
0	1	False	0.5π	0	0	0	0
0	1	True	0	−1	+1	±1	0
1	0	False	0.5π	−1	+1	±1	0
1	0	True	0	+1	−1	0	±1
1	1	False	0.2π	+1	−1	0	±1
1	1	True	0	+1	−1	0	±1

**Table 2:** CPU parameters.

CPU	
Model	Intel Core i5-6500
Cores	4
Cache	6 MB
Threads	4
Instruction Set	64 Bit
Base Frequency	3.2 GHz

**Table 3:** Selected results.

$p_{LO}$	0.8	0.7	0.6	0.5	0.4	0.3	0.2	0.1
Dev	1.053777	1.044784	0.932192	0.867456	0.728421	0.893458	0.91159	0.961345

**Table 4:** Selected results of 37 test instances.

Instance	$f_{\mathrm{ref}}$	$f_{{\mathrm{best}}_{\mathrm{init}}}$	$f_{\mathrm{best}}$	$f_{{\mathrm{best}}_{\mathrm{avr}}}$	Iter	${\mathrm{Iter}}_{\mathrm{av}}$	$T_{\mathrm{avr}}$	Dev[%]
BUR26A	5426670	5635476	5463327	5487776	3041	4451	77880	0.67
BUR26B	3817852	3983271	3856650	3861719	2221	3413	74712	1.01
BUR26C	5426795	5688890	5464668	5494245	3289	6847	77616	0.69
BUR26D	3821225	4021377	3860910	3873609	1963	3845	79332	1.03
BUR26E	5386879	5674734	5434198	5457173	821	5343	83952	0.87
BUR26F	3782044	3981808	3816849	3833165	618	5080	72468	0.92
BUR26G	10117172	10448706	10246167	10272730	168	1652	78408	1.27
BUR26H	7098658	7474339	7177185	7200100	755	3165	90288	1.10
CHR22A	6156	9442	7610	7643	3179	5976	54516	23.61
CHR22B	6194	10124	7486	7650	199	1719	58476	20.85
ESC32A	130	314	216	227.5	429	4427	95700	66.15
ESC32B	160	352	224	255	5038	6767	116952	40.00
ESC32C	642	818	642	645.5	1768	5691	111012	0.00
ESC32D	200	294	220	222.5	2949	6301	101640	10.00
ESC32E	2	6	2	2	1	5	9824	0.00
ESC32F	2	10	2	2	1	2	101112	0.00
ESC32G	6	18	6	6	6	39	113520	0.00
ESC32H	438	586	464	478.5	1919	5170	92532	5.93
ESC64A	116	228	142	148	7804	8837	216876	22.41
KRA30A	88900	121740	103150	104897.5	2758	4957	102432	16.02
KRA30B	91420	123640	103640	107377.5	2440	6106	109956	13.36
LIPA30A	13178	13679	13570	13574.5	2559	5383	101904	2.97
LIPA30B	151426	189936	181552	183420.3	3021	6931	95304	19.89
LIPA40A	31538	32551	32309	32327.25	1448	3734	108768	2.44
LIPA40B	476581	605311	585031	591119	186	4934	111540	22.75
LIPA50A	62093	63692	63477	63505.75	2221	3869	130416	2.22
LIPA50B	1210244	1531788	1498885	1504987	1404	5586	155364	23.84
LIPA60A	107218	109696	109370	109401.5	6159	6484	189024	2.00
LIPA60B	2520135	3209806	3170192	3173960	1347	5068	212520	25.79
SKO42	15812	18968	18070	18108.5	3127	6995	121968	14.28
SKO49	23386	27942	26672	26720.5	58	3075	151404	14.05
SKO56	34458	40586	39146	39313.5	2939	5513	169224	13.60
STE36A	9526	17274	12872	13915	1280	5315	114048	35.12
STE36B	15852	54246	28996	33159.5	3449	6034	114312	82.91
STE36C	8239110	14268786	11293534	11529941	5108	6986	105072	37.07
THO30	149936	192394	173998	175704	1279	4294	80916	16.04
THO40	240516	312066	285288	290608	1240	6486	112860	18.61
Avr								15.12

**Table 5:** Best results of 37 test instances.

Instance	$f_{\mathrm{ref}}$	$f_{\mathrm{best}}$	Dev[%]	${\mathrm{Dev}}_{\mathrm{GA}}$	${\mathrm{Dev}}_{\mathrm{GASA}}$	Instance	$f_{\mathrm{ref}}$	$f_{\mathrm{best}}$	Dev[%]	${\mathrm{Dev}}_{\mathrm{GA}}$	${\mathrm{Dev}}_{\mathrm{GASA}}$
BUR26A	5426670	5458907	0.59	0.00000	0.00000	KRA30A	88900	90200	1.46	0.00000	0.00000
BUR26B	3817852	3856650	1.01	0.00000	0.00000	KRA30B	91420	103640	13.36	0.00000	0.00000
BUR26C	5426795	5464668	0.69	0.00010	0.00010	LIPA30A	13178	13570	2.97	0.00000	0.00000
BUR26D	3821225	3860910	1.03	0.00080	0.00010	LIPA30B	151426	181552	19.89	0.00000	0.00000
BUR26E	5386879	5386954	0.01	0.00002	0.00002	LIPA40A	31538	32309	2.44	0.00000	0.00000
BUR26F	3782044	3816849	0.92	0.00000	0.00000	LIPA40B	476581	497926	4.47	0.00000	0.00000
BUR26G	10117172	10246167	1.27	0.00030	0.00230	LIPA50A	62093	62866	1.24	0.00000	0.95180
BUR26H	7098658	7177185	1.10	0.00000	0.00000	LIPA50B	1210244	1344530	11.09	0.00000	0.00000
CHR22A	6156	6290	2.17	0.32490	0.00000	LIPA60A	107218	109370	2.00	0.76110	0.79931
CHR22B	6194	7486	20.85	2.71230	1.32390	LIPA60B	2520135	2980033	18.24	0.00020	0.00020
ESC32A	130	134	3.07	3.07690	3.07690	SKO42	15812	18070	14.28	0.00000	0.10119
ESC32B	160	168	5.00	5.00000	5.00000	SKO49	23386	26672	14.05	0.24800	0.11970
ESC32C	642	642	0.00	0.00000	0.00000	SKO56	34458	39146	13.60	0.29600	0.20900
ESC32D	200	220	10.00	0.00000	0.00000	STE36A	9526	9800	2.87	0.25190	0.00000
ESC32E	2	2	0.00	0.00000	0.00000	STE36B	15852	16424	3.60	0.80750	0.00000
ESC32F	2	2	0.00	0.00000	0.00000	STE36C	8239110	8419654	2.19	0.00000	0.19970
ESC32G	6	6	0.00	0.00000	0.00000	THO30	149936	173998	16.04	0.22940	0.29480
ESC32H	438	464	5.93	0.00000	0.00000	THO40	240516	285288	18.61	0.17380	0.16050
ESC64A	116	142	22.4	0.00000	0.00000						
$Dev_{avr}$									6.45	0.37	0.32

## References

[1] Burkard R., Dell’Amico M., Martello S. (2009). Assignment Problems.

[2] Sahni S., Gonzalez T. (1976). P-complete approximation problems. J. ACM.

[3] Drezner Z., Laporte G., Nickel S., Saldanha da Gama F. (2015). The quadratic assignment problem. Location Science.

[4] Loiola E.M., De Abreu N.M.M., Boaventura-Netto P.O., Hahn P., Querido T. (2007). A survey for the quadratic assignment problem. Eur. J. Oper. Res..

[5] Misevicius A. (2005). A tabu search algorithm for the quadratic assignment problem. Comput. Optim. Appl..

[6] Battiti R., Tecchiolli G. (1994). Simulated annealing and tabu search in the long run: A comparison on {QAP} tasks. Comput. Math. Appl..

[7] Drezner Z. (2005). The extended concentric tabu for the quadratic assignment problem. Eur. J. Oper. Res..

[8] Wilhelm M.R., Ward T.L. (1987). Solving quadratic assignment problems by ‘Simulated Annealing’. IIE Trans..

[9] Misevičius A. (2003). A modified simulated annealing algorithm for the quadratic assignment problem. Informatica.

[10] Dorigo M., Di Caro G., Gambardella L.M. (1999). Ant algorithms for discrete optimization. Artif. Life.

[11] Lv C., Zhao H., Yang X. Particle swarm optimization algorithm for quadratic assignment problem. Proceedings of the 2011 International Conference on Computer Science and Network Technology (ICCSNT).

[12] Liu H., Abraham A., Zhang J., Saad A., Dahal K., Sarfraz M., Roy R. (2007). A particle swarm approach to quadratic assignment problems. Soft Computing in Industrial Applications.

[13] Chmiel W., Szwed P. (2016). Bees algorithm for the quadratic assignment problem on CUDA platform. Man–Machine Interactions 4.

[14] Chmiel W., Kadłuczka P., Kwiecień J., Filipowicz B. (2017). A comparison of nature inspired algorithms for the quadratic assignment problem. Bull. Pol. Acad. Sci.-Tech..

[15] Tate D.M., Smith A.E. (1995). A genetic approach to the quadratic assignment problem. Comput. Oper. Res..

[16] Drezner Z. (2003). A new genetic algorithm for the quadratic assignment problem. INFORMS J. Comput..

[17] Drezner Z. (2005). Compounded genetic algorithms for the quadratic assignment problem. Oper. Res. Lett..

[18] Drezner Z. (2008). Extensive experiments with hybrid genetic algorithms for the solution of the quadratic assignment problem. Comput. Oper. Res..

[19] Talbi H., Batouche M., Draa A. (2007). A quantum-inspired evolutionary algorithm for multiobjective image segmentation. Int. J. Math. Phys. Eng. Sci..

[20] Lin D.Y., Waller S. (2009). A quantum-inspired genetic algorithm for dynamic continuous network design problem. Transp. Lett..

[21] Xing H., Ji Y., Bai L., Liu X., Qu Z., Wang X. (2009). An adaptive-evolution-based quantum-inspired evolutionary algorithm for QoS multicasting in IP/DWDM networks. Comput. Commun..

[22] Wang L., Wu H., Tang F., Zheng D.Z., Huang D.S., Zhang X.P., Huang G.B. (2005). A hybrid quantum-inspired genetic algorithm for flow shop scheduling. Advances in Intelligent Computing.

[23] Li B.B., Wang L. (2006). A hybrid quantum-inspired genetic algorithm for multi-objective scheduling. ICIC Intelligent Computing.

[24] Gu J., Gu M., Cao C., Gu X. (2010). A novel competitive co-evolutionary quantum genetic algorithm for stochastic job shop scheduling problem. Comput. Oper. Res..

[25] Wu X., Wu S. (2017). An elitist quantum-inspired evolutionary algorithm for the flexible job-shop scheduling problem. J. Intell. Manuf..

[26] Vlachogiannis J.G., Lee K.Y. (2008). Quantum-inspired evolutionary algorithm for real and reactive power dispatch. IEEE Trans. Power Syst..

[27] Wang L., Li L.P. (2010). An effective hybrid quantum-inspired evolutionary algorithm for parameter estimation of chaotic systems. Expert Syst. Appl..

[28] Luo Z., Wang P., Li Y., Zhang W., Tang W., Xiang M. (2008). Quantum-inspired evolutionary tuning of SVM parameters. Prog. Nat. Sci..

[29] Gupta S., Mittal S., Gupta T., Singhal I., Khatri B., Gupta A., Kumar N. (2017). Parallel quantum-inspired evolutionary algorithms for community detection in social networks. Appl. Soft Comput..

[30] Misevičius A., Rubliauskas D. (2009). Testing of hybrid genetic algorithms for structured quadratic assignment problems. Informatica.

[31] Misevicius A., Guogis E., Skersys T., Butleris R., Butkiene R. (2012). Computational study of four genetic algorithm variants for solving the quadratic assignment problem. Information and Software Technologies.

[32] Benlic U., Hao J.K. (2015). Memetic search for the quadratic assignment problem. Expert Syst. Appl..

[33] Lalla-Ruiz E., Expósito-Izquierdo C., Melián-Batista B., Moreno-Vega J.M. (2016). A hybrid biased random key genetic algorithm for the quadratic assignment problem. Inf. Process. Lett..

[34] Luong N.H., Poutré H.L., Bosman P.A. (2018). Multi-objective Gene-pool optimal mixing evolutionary algorithm with the interleaved multi-start scheme. Swarm Evol. Comput..

[35] Atencio F., Neira D. (2016). A sule’s method initiated genetic algorithm for solving QAP formulation in facility layout design: A real world application. J. Theor. Appl. Inf. Technol..

[36] Ahuja R.K., Orlin J.B., Tiwari A. (2000). A greedy genetic algorithm for the quadratic assignment problem. Comput. Oper. Res..

[37] Tosun U. (2014). A new recombination operator for the genetic algorithm solution of the quadratic assignment problem. Procedia Comput. Sci..

[38] Metlicka M., Davendra D. (2015). Chaos driven discrete artificial bee algorithm for location and assignment optimisation problems. Swarm Evol. Comput..

[39] Sghir I., Hao J.K., Jaafar I.B., Ghedira K. (2015). A multi-agent based optimization method applied to the quadratic assignment problem. Expert Syst. Appl..

[40] Duman E., Uysal M., Alkaya A.F. (2012). Migrating birds optimization: A new metaheuristic approach and its performance on quadratic assignment problem. Inf. Sci..

[41] Oliveira S., Hussin M.S., Roli A., Dorigo M., Stützle T. Analysis of the population-based ant colony optimization algorithm for the TSP and the QAP. Proceedings of the 2017 IEEE Congress on Evolutionary Computation (CEC).

[42] Hafiz F., Abdennour A. (2016). Particle swarm algorithm variants for the quadratic assignment problems—A probabilistic learning approach. Expert Syst. Appl..

[43] Dokeroglu T., Cosar A. (2016). A novel multistart hyper-heuristic algorithm on the grid for the quadratic assignment problem. Eng. Appl. Artif. Intell..

[44] Tasgetiren M.F., Pan Q.K., Ozturkoglu Y., Cotur O.K. Variable block insertion heuristic for the quadratic assignment problem. Proceedings of the 2017 IEEE Congress on Evolutionary Computation (CEC).

[45] Yuan Y., Ong Y.S., Gupta A., Tan P.S., Xu H. Evolutionary multitasking in permutation-based combinatorial optimization problems: Realization with TSP, QAP, LOP, and JSP. Proceedings of the 2016 IEEE Region 10 Conference (TENCON).

[46] Aksan Y., Dokeroglu T., Cosar A. (2017). A stagnation-aware cooperative parallel breakout local search algorithm for the quadratic assignment problem. Comput. Ind. Eng..

[47] Acan A., Ünveren A. (2015). A great deluge and tabu search hybrid with two-stage memory support for quadratic assignment problem. Appl. Soft Comput..

[48] Zhang G. (2011). Quantum-inspired evolutionary algorithms: A survey and empirical study. J. Heuristics.

[49] Elshafei A.N. (1977). Hospital layout as a quadratic assignment problem. J. Oper. Res. Soc..

[50] Dickey J., Hopkins J. (1972). Campus building arrangement using topaz. Transp. Res..

[51] Duman E., Or I. (2007). The quadratic assignment problem in the context of the printed circuit board assembly process. Comput. Oper. Res..

[52] Han K.H., Kim J.H. (2002). Quantum-inspired evolutionary algorithm for a class of combinatorial optimization. IEEE Trans. Evol. Comput..

[53] Hey T. (1999). Quantum computing: An introduction. Comput. Control Eng. J..

[54] Gu J., Gu X., Gu M. (2009). A novel parallel quantum genetic algorithm for stochastic job shop scheduling. J. Math. Anal. Appl..

[55] Lahoz-Beltra R. (2016). Quantum genetic algorithms for computer scientists. Computers.

[56] Misevićius A., Kilda B. (2005). Comparison of crossover operators for the quadratic assignment problem. Inf. Technol. Control.

[57] Chmiel W., Kadłuczka P., Packanik G. (2009). Performance of swarm algorithms for permutation problems. Automatyka.

[58] Chmiel W., Kadluczka P. A multi-phase diversification method of population in the evolutionary algorithm. Proceedings of the XVI National Conference on Discrete Process Automation.

